# Modeling UBC intrinsic excitability

**DOI:** 10.1186/1471-2202-12-S1-P322

**Published:** 2011-07-18

**Authors:** Sathyaa Subramaniyam, Paola Perin, Sergio Solinas, Egidio D’Angelo

**Affiliations:** 1 Department of Physiology, University of Pavia, Via Forlanini 6, I-27100, Pavia, Italy; 2 Consorzio Interuniversitario per le Scienze Fisiche della MateriaÂ (CNISM), Via Bassi 6,Â I-27100 Pavia, Italy; 3 Brain Connectivity Center, Istituto Neurologico IRCCS C. Mondino, Via Mondino 2, I-27100 Pavia, Italy

## 

Unipolar brush cells (UBCs) are excitatory glutamatergic interneurons of the cerebellar granular layer receiving both primary and secondary vestibular inputs through mossy fibers (excitatory input) and Golgi axon (inhibitory input). The brush like structure of the dendrite allows to form a giant synapses in the glomerulus and to produce an all or none post synaptic response with short delay and protracted kinetics. The excitable response of UBCs can be either a tonic discharge or a high-frequency burst of action potentials. When injected with progressively increasing depolarizing currents from a negative membrane potential, the UBC generates a burst sustained by a calcium spike (Figure 1, +20pA) and then a protracted discharge with shorter latency and spike frequency adaption (Figure 1, +25pA). The intrinsic excitability of UBCs is determined by an H current and by Low Voltage activated and High Voltage activated calcium currents [2,3]. Fast inactivating T-type Calcium channels generate low-threshold spikes and L-type Calcium channel sustain tonic firing. The H current (activated between -60mV and -80mV) produces a slow hyperpolarization characterized by a “sag” in response to a hyperpolarizing step (Figure 1, -16pA) and an afterhyperpolarization at the end of a depolarizing step.

**Figure 1:**
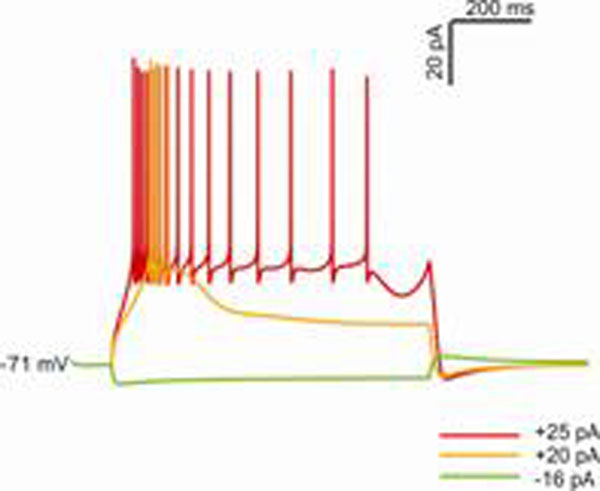
Excitable response of the UBC model to step-current injection.

## Conclusions

Here we present a biologically realistic multi-compartmental mathematical model of the UBC realized with the NEURON simulator. According to literature [1-4], ionic channels are distributed among compartments (soma, dendrite, and axon). The model can reproduce the excitable properties of UBCs in current-clamp and voltage-clamp modes. Attempts at modeling the response to mossy fiber inputs are ongoing. This model confirms the primary role of the aforementioned currents in UBC’s electroresponsiveness. The model will also be a valuable tool for investigating the UBC’s function in the cerebellar network.
